# Plasma Heme Scavengers Alpha-1-Microglobulin and Hemopexin as Biomarkers in High-Risk Pregnancies

**DOI:** 10.3389/fphys.2019.00300

**Published:** 2019-04-04

**Authors:** Grigorios Kalapotharakos, Katja Murtoniemi, Bo Åkerström, Esa Hämäläinen, Eero Kajantie, Katri Räikkönen, Pia Villa, Hannele Laivuori, Stefan R. Hansson

**Affiliations:** ^1^Department of Clinical Sciences Lund, Skåne University Hospital, Lund, Sweden; ^2^Department of Obstetrics and Gynecology, Lund University, Lund, Sweden; ^3^Medical and Clinical Genetics, University of Helsinki and Helsinki University Hospital, Helsinki, Finland; ^4^Department of Obstetrics and Gynecology, Turku University Hospital and University of Turku, Turku, Finland; ^5^Division of Infection Medicine, Department of Clinical Sciences, Lund University, Lund, Sweden; ^6^HUSLAB, Helsinki University Hospital, Helsinki, Finland; ^7^Department of Clinical Chemistry, University of Helsinki, Helsinki, Finland; ^8^National Institute for Health and Welfare, Helsinki, Finland; ^9^Children’s Hospital, University of Helsinki and Helsinki University Hospital, Helsinki, Finland; ^10^Research Unit for Pediatrics, Dermatology, Clinical Genetics, Obstetrics and Gynecology, Medical Research Center Oulu, Oulu University Hospital and University of Oulu, Oulu, Finland; ^11^Department of Psychology and Logopedics, Faculty of Medicine, University of Helsinki, Helsinki, Finland; ^12^Department of Obstetrics and Gynecology, University of Helsinki and Helsinki University Hospital, Helsinki, Finland; ^13^Institute for Molecular Medicine Finland, Helsinki Institute of Life Science, University of Helsinki, Helsinki, Finland; ^14^Department of Obstetrics and Gynecology, Tampere University Hospital, Tampere, Finland; ^15^Faculty of Medicine and Life Sciences, University of Tampere, Tampere, Finland

**Keywords:** preeclampsia, heme, hemopexin, alpha-1 microglobulin, aspirin

## Abstract

Women with established preeclampsia (PE) have increased plasma concentration of free fetal hemoglobin. We measured two hemoglobin scavenger system proteins, hemopexin (Hpx) and alpha-1-microglobulin (A1M) in maternal plasma using enzyme-linked immunosorbent assay during the late second trimester of pregnancy in women with high and low risk of developing PE. In total 142 women were included in nested case-control study: 42 women diagnosed with PE and 100 controls (49 randomly selected high-risk and 51 low-risk controls). The concentration of plasma A1M in high-risk controls was higher compared to low-risk controls. Women with severe PE had higher plasma A1M levels compared to women with non-severe PE. In conclusion, the concentration of plasma A1M is increased in the late second trimester in high-risk controls, suggesting activation of endogenous protective system against oxidative stress.

## Introduction

Preeclampsia (PE) is a relatively common hypertensive disorder in pregnancy, affecting 4,6% of pregnancies worldwide (Abalos et al., 2013). The specific etiology of PE is, however, still not completely described. According to the most well-accepted model, PE is a two-stage disorder (Redman, 1991; Redman et al., 2014). The abnormal modification of the spiral arteries during placental development is thought to be the initial stage leading to reduced utero-placental perfusion and increased oxidative stress that in turn causes placental damage. Circulating toxic factors derived from the placenta cross the blood-placenta barrier and leak into the maternal circulation where they in turn trigger an inflammatory response and general endothelial damage. As a consequence of that, general organ damage develops, which leads to the typical manifestations of PE after 20th week of gestation, including hypertension, edema and proteinuria. Circulating syncytiotrophoblast microvesicles (Smarason et al., 1996), free fetal DNA (Zhong et al., 2002), cytokines (Hamai et al., 1997) and antiangiogenic factors (Powe et al., 2011) have been postulated as endothelial toxic factors derived from the fetus and the placenta. However, we still lack a full explanation on how placental damage leads to distinct maternal and fetal manifestations that occur either during early pregnancy or late pregnancy, so called early onset PE and late-onset PE. Early onset PE is linked to poor placentation while the late-onset is more determined by maternal risk factors such as obesity, diabetes mellitus and chronic hypertension, which are associated with a higher pre-pregnancy level of vascular inflammation (Roberts and Redman, 1993; Ness and Roberts, 1996).

Extracellular fetal hemoglobin (HbF) has been introduced in a series of earlier studies and suggested to have a crucial role in the etiology of PE (Hansson et al., 2013). Increased synthesis of HbF in the placenta was indicated by an up-regulation of the HbF genes and there was an accumulation of extracellular HbF in the vascular lumen of PE placenta (Centlow et al., 2008). Extracellular HbF induces oxidative stress by formation of reactive oxygen species resulting in damage to the blood-placenta barrier and leakage of extracellular HbF into the maternal circulation (May et al., 2011). As a consequence, plasma concentration of extracellular HbF has been shown to be increased in maternal plasma as early as the first trimester in women who later develop PE (Anderson et al., 2011). Increased plasma levels in the late third trimester has been shown to correlate to the maternal blood pressure (Anderson et al., 2018).

There are several defense mechanisms which protect against the harmful effects of extracellular hemoglobin (Hb). Haptoglobin (Hp) is the most important protective scavenger protein that binds extracellular Hb in plasma resulting in a complex that in turn is cleared via CD163 receptors on macrophages (Kristiansen et al., 2001; Schaer et al., 2006). Hemopexin (Hpx) has a complementary role to bind extracellular heme that is released as a metabolite when Hb is degraded by the rate-limiting enzyme heme-oxygenase (HO-1) (Nielsen et al., 2010; Tolosano et al., 2010). The resulting complex is cleared from the circulation by liver parenchymal cells via receptor-mediated endocytosis involving CD91/LRP1. Alpha-1-microglobulin (A1M) is another component of the heme scavenger system (Akerstrom and Gram, 2014). It is a lipocalin with heme-binding properties as well as being an antioxidant due to radical-scavenging and reductase properties. In a series of studies (Gram et al., 2015; Anderson et al., 2018), it has been shown that the plasma levels of Hp and Hpx are reduced, suggesting that in cases where the maternal endogenous protection system against extracellular HbF is overwhelmed, PE becomes clinically manifest. Cellular A1M expression of A1M is upregulated by increased oxidative stress and Hb/heme exposure (Olsson et al., 2007) and previous investigations have shown increased circulating plasma levels of A1M in women pregnant with PE (Olsson et al., 2010; Anderson et al., 2011) consistent with high circulating levels of Hb, heme and oxidants in this disease.

In the present study, we analyzed the plasma levels of A1M and Hpx in order to further understand the dynamics of these components of the Hb/heme scavenger system in the second trimester in women with high and low risk of developing PE. The cohort of patients were stratified according to known maternal risk factors, intervention with acetylsalicylic acid (ASA) as well as neonatal outcome.

## Materials and Methods

### Study Population

The present nested case-control study is a part of the multidisciplinary “Prediction and Prevention of Pre-eclampsia and Intrauterine Growth Restriction” (PREDO) project. Women with known risk factors for PE were prospectively recruited between September 2005 and June 2009 at ten participating maternity clinics in Finland. The ethics Committee at the Helsinki and Uusimaa Hospital District approved the study and written informed consent was obtained from all participants.

In total 142 women were included in this study: 42 women diagnosed with PE and 100 controls (49 randomly selected high-risk and 51 low-risk controls). Seven women with PE participated in the ASA trial (part of the PREDO project), as well, and were treated with low dose acetylsalicylic acid (mini-ASA 100mg/d) starting before 14th week of gestation. Three women who were taking mini-ASA and did not develop PE, were included as controls for this sub-group. The inclusion and exclusion criteria are described in Supplementary Table S1.

Preeclampsia was defined as a systolic blood pressure ≥140 mmHg and/or a diastolic blood pressure ≥90 mmHg occurring after 20th weeks of gestation combined with a urinary 24-h protein excretion of ≥0.3 g or the dipstick equivalent in two consecutive measurements. Severe PE was defined as systolic blood pressure ≥160 mmHg and/or diastolic blood pressure ≥110 mmHg and/or proteinuria ≥5 g/24 h. Small for gestational age (SGA) was defined as a birthweight ≤minus 2 SDs.

All participants had their first visit at 12^+0^–14^+0^weeks of gestation. Uterine artery blood flow was measured with Doppler ultrasound examination. Gestational age was confirmed by crown-rump length measurement. The first trimester mean arterial pressure (MAP) was calculated with the equation: MAP = diastolic blood pressure + (systolic blood pressure – diastolic blood pressure)/3.

Fasting blood samples were collected in all three trimesters. Plasma was separated within an hour by centrifugation and stored in -80^°^C until analysis. In the present study we determined serum A1M and Hpx concentrations from samples drawn at 26^+0^ to 28^+0^weeks of gestation.

### Hemopexin ELISA

The Hpx concentrations were measured with a Human Hemopexin ELISA Kit from Genway Biotech Inc. The analysis was performed according to manufacturer’s instructions and the absorbance was read at 450 nm using a Wallac 1420 Multilabel Counter.

### A1M ELISA

The A1M concentrations were measured with an in-house A1M ELISA. Flat-bottom ninety six-well microtiter plates were coated with mouse monoclonal anti-A1M antibodies (clone 35.14) by incubation overnight at +4°C under sealing film with 100 μl/well of a 5 μg/ml-solution in PBS. After washing three times with PBS + 0.05% tween-20, 100 μl of human urinary A1M reference standard samples (1.56 – 100 ng/ml in PBS + 0.05% tween-20) or unknown plasma samples (diluted 1000× with PBS + 0.05% tween-20) were added to the wells and incubated under sealing film for 1 h at room temperature, darkness and rotational shaking 250–500 rpm. After washing three times with PBS + 0.05% tween-20, 100 μl/well of the detection antibody solution was added (horse radish peroxidase-coupled mouse monoclonal anti-A1M antibody clone 57.10; 5 ng/ml in PBS + 0.05% tween-20) and incubated under sealing film for 1 h at room temperature, darkness and rotational shaking 250–300 rpm. After washing three times with PBS + 0.05% tween-20, 100 μl/well of TMB substrate (SureBlue^TM^ TMB Microwell Peroxidase Substrate, KPL cat. no. 50-00-04) was added, sealed, and incubated 20 min without shaking, and the reaction was stopped by adding 100 μl/well of 1 M sulfuric acid. Absorbance at 450 nm was read in a Wallac 1420 Multilabel Counter (Perkin Elmer Life Sciences). The monoclonal anti-A1M antibodies were prepared against human urinary A1M by Agrisera AB (Vännäs, Sweden). Human urinary A1M was prepared in our lab as described (Akerstrom et al., 1995).

### Statistical Analysis

Statistical analyses were performed using SPSS version 25.0 statistic software package. Normally distributed data were analyzed using one-way ANOVA followed by Tukey’s *post hoc* tests. Kruskal-Wallis and Mann-Whitney test were used in case the data were not normally distributed and Bonferroni corrections were used in *post hoc* comparisons. Statistical significance was defined as *p* < 0.05.

## Results

Patient demographics and clinical characteristics are shown in Table 1, 2. Women affected by PE had higher body mass index (29,3 kg/m^2^) compared to controls (23,7 kg/m^2^). There were less primiparas among women affected by PE compared to controls. There were less women with a previous pregnancy complicated by PE among controls compared to women affected by PE.

**Table 1:** Demographics of patients and controls.

	Women not	Women
	affected by	affected by
	PE (n = 100)	PE (n = 42)	*P*-value	OR^a^	95% CI	
					Lower	Upper
Age, years (SD)^b^	31.3 (4.4)	31.6 (5.2)	0.74	1.01	0.94	1.10
BMI, pre-pregnancy, kg/m^2^ (IQR)^c^	23.7 (7.3)	29.3 (10.6)	<0.01	1.09	1.03	1.15
Primiparous, n (%)	42 (42.0)	10 (23.8)	0.04	1.53	1.01	2.30
Infertility treatment, n (%)	11 (11.0)	5 (11.9)	0.77	1.19	0.38	3.70
Chronic disease, n (%)	37 (37.0)	22 (52.4)	0.67	0.18	0.80	3.26
Education, n (%)						
Elementary or less^d^	0 (0.0)	3 (7.9)	0.02			
High school or vocational school	22 (22.0)	8 (19.0)	0.64	0.90	0.36	2.26
Intermediate	32 (32.0)	19 (45.2)	0.83	0.90	0.36	2.26
University	40 (40.0)	7 (17.0)	0.01	0.32	0.13	0.79
Prior preeclampsia	14 (14.0)	21 (50.0)	<0.01	0.16	0.07	0.37
SGA in previous pregnancy	9 (9.0)	4 (9.5)	0.92	0.94	0.27	3.24
Chronic hypertension	14 (14.0)	10 (23.8)	0.16	0.52	0.21	1.29
Prior GDM	4 (4.0)	4 (9.5)	0.32	0.50	0.13	1.96
BMI ≥ 30 kg/m^2^	22 (22.0)	16 (38.1)	0.05	0.46	0.21	1.00
Prior fetus mortuus^d^	2 (2.0)	1 (2.4)	1.00			


*^*a*^binary logistic regression, ^*b*^mean, ^*c*^median, ^*d*^fisher’s exact test; PE = preeclampsia, OR = odds ratio, CI = confidence interval, SD = standard deviation, BMI = body mass index. There was one type I diabetes mellitus and one Sjögren’s syndrome in women who did not develop preeclampsia. There was not systemic lupus erythematosus in either group.*

**Table 2:** Clinical characteristics of patients and controls.

	Women not	Women
	affected by	affected by			95%	CI
	PE *n* = 100	PE *n* = 42	*P*-value	OR	Upper	Lower
Weight change during pregnancy, kg/m^2^	14.5 (7.8)	12.0 (6.6)	0.18	0.95	0.88	1.02
Gestational diabetes, n (%)	17 (17.0)	12 (28.6)	0.12	0.51	0.22	1.20
I trimester mean arterial pressure, mmHg	89.7 (15.7)	98.8 (13.8)	<0.01	1.07	1.04	1.11
I trimester mean uterine artery PI	0.99 (0.35)	1.25 (0.47)	<0.01	14.64	3.45	62.04
Highest mean arterial pressure, mmHg	100.0 (13.5)	128.5 (15.2)	<0.01	1.16	1.11	1.22
Gestational weeks at birth	40.3 (1.9)	38.4 (3.2)	<0.01	0.53	0.41	0.70
Apgar score at 5 min	9 (1)	9 (2)	0.32	0.80	0.51	1.25
Umbilical artery pH	7.25 (0.13)	7.25 (0.13)	0.24	0.08	<0.01	5.57
Newborn birthweight, g	3590 (651)	3109 (1259)	<0.01	0.999	0.998	0.999
Placental weight, g	605 (175)	540 (173)	<0.01	0.995	0.992	0.998


*Continuous variables are presented as median values with interquartile range in parenthesis. n=number of cases, PE=preeclampsia.*

**FIGURE 1:**
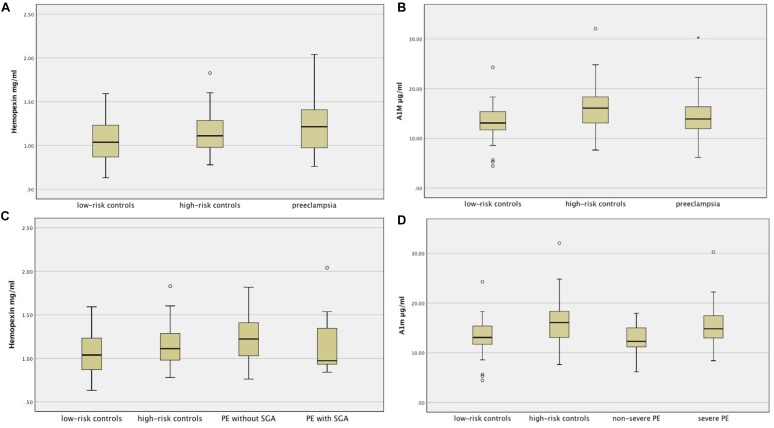
**(A)**: Distribution of hemopexin across groups. **(B)**: Distribution of A1M across groups. **(C)**: Distribution of hemopexin across subgroups of PE, high- and low-risk controls. **(D):** Distribution of A1M across subgroups of PE, high- and low-risk controls.

The distributions of plasma Hpx and A1M across the groups are shown in Figure 1A,B. Pairwise comparisons using significance values adjusted by the Bonferroni correction for multiple tests revealed that the concentration of Hpx in women with PE was higher compared to low-risk normotensive women (median concentration 1.21 mg/ml versus 1.04 mg/ml, *p* = 0.014). There was no significant difference in Hpx concentration between women with PE and high-risk normotensive women (median concentration 1.11 mg/ml) or between high-risk and low-risk normotensive women. The concentration of A1M in high-risk controls was higher compared to low-risk controls (median concentration 16.08 μg/ml vs. 13.09 μg/ml, *p* = 0.002). There was no significant difference in A1M concentration between women with PE and high-risk controls or between women with PE and low-risk controls.

The concentrations of plasma Hpx and A1M in women with PE are shown in Table 3. We found no difference in Hpx or A1M concentrations between women with PE who gave birth to SGA infant and women with PE who gave birth to non-SGA infant. The concentration of A1M in women with severe PE was higher compared to women with non-severe PE. The distributions of Hpx and A1M across the subgroups of PE, high- and low-risk controls are shown in Figure 1C,D.

**Table 3:** Values of A1M and hemopexin across subgroups of women with PE.

		Hemopexin (mg/ml)	A1M (μg/ml)
PE onset	late onset (*n* = 31) versus early onset (*n* = 11)	1.22 (0.32) versus 1.16 (0.64), *p* = 0.66	13.34 (3.26) versus 16.66 (3.96), *p* = 0.07
SGA	no (*n* = 34) versus yes (*n* = 8)	1.22 (0.41) versus 0.97 (0.52), *p* = 0.183	13.20 (3.84) versus 16.85 (5.83), *p* = 0.11
PE severity	mild (*n* = 21) versus severe (*n* = 21)	1.22 (0.56) versus 1.21 (0.44), *p* = 0.84	12.30 (4.46) versus 14.83 (5.23), *p* = 0.04
ASA	no (*n* = 35) versus yes (*n* = 7)	1.20 (0.43) versus 1.31 (0.51), *p* = 0.16	14.24 (4.42) versus 12.13 (3.10), *p* = 0.30


*Continuous variables are presented as median values with interquartile range in parenthesis. n=number of cases.*

Seven PE women received mini-ASA during the pregnancy and one of them gave birth to SGA infant. Thirty-five PE women did not receive mini-ASA and eight of them gave birth to SGA infant. Women who received ASA had higher Hpx concentration compared to women who did not receive ASA (median concentration 1,28 mg/ml versus 1,09 mg/ml, *p* = 0,025).

## Discussion

To the best of our knowledge, this is the first study evaluating the levels of maternal plasma Hpx and A1M in the late second trimester in PE women as well as in high- and low-risk controls without PE. The strength of the study is a carefully characterized cohort, where both women with predetermined risk factors for PE and a low-risk reference group were prospectively recruited (Girchenko et al., 2017).

Our analysis shows a significantly higher plasma A1M concentration in high-risk controls compared to low-risk controls, while there was no significant difference in concentration of A1M between PE women and controls. In previous studies (Olsson et al., 2010; Anderson et al., 2011, 2016; Gram et al., 2015), the concentration of A1M in PE women was increased compared to controls when it was analyzed during the first trimester and third trimester, 24 h prior to delivery. In the aforementioned studies, normotensive pregnant women had been studied as a single undivided group, irrespective of risk factors for PE. However, it is reasonable to hypothesize that high-risk women are a distinct group characterized by increased oxidative stress compared to low-risk women.

In the present cohort, pre-pregnancy obesity was among the criteria conferring high-risk status to normotensive pregnant women. Obesity is associated with systemic oxidative stress (Furukawa et al., 2004). It can therefore be assumed that in high-risk controls, the endogenous protection system against oxidative stress is activated. The housekeeping protein A1M is an extravascular scavenger and tissue repair protein which has an important role in cleaning oxidative radicals and heme. It is upregulated during oxidative stress in general and by hemolysis specifically (Akerstrom and Gram, 2014). Thus, our results may suggest that increased oxidative stress is present in high-risk normotensive pregnant women compared to low-risk controls in late second trimester. A serious weakness with this argument, however, is that we have not included evaluation of oxidative stress markers. Evaluation of oxidative stress markers and reactive oxidative species measurements need to be undertaken before the association between A1M and oxidative stress in high-risk normotensive women is more clearly understood. Although A1M has not been shown to be an acute-phase protein, increased levels of A1M in high-risk normotensive women as a result of chronic inflammation related to obesity might be another explanation and needs to be clarified in further studies (Akerstrom and Gram, 2014).

Interestingly, the median plasma A1M concentration in high-risk normotensive women was increased compared to PE women, although the difference was not statistically significant. Higher concentration of A1M may confer its protective effect ameliorating the clinical impact of oxidative stress and preventing the development of PE in high-risk controls who remain asymptomatic. In line with this assumption, intravenous administration of a recombinant version of A1M in PE animal models has been successful in eliminating or at least significantly reducing the manifestations of preeclampsia (Sverrisson et al., 2014; Nääv et al., 2015; Gunnarsson et al., 2017). One can therefore speculate that the administration of exogenous A1M might have a similar effect in humans and PE women also.

Recent evidence suggests that extracellular HbF is elevated in the fetal circulation of pregnancies complicated by fetal growth restriction (Brook et al., 2018). We expect that among SGA infants in our cohort there are both constitutionally small infants and infants that have not reached their growth potential because of placental dysfunction. In the latter case, we expect that extracellular HbF in the fetal circulation is elevated causing, as we have previously shown (Centlow et al., 2008; May et al., 2011), damage to blood-placenta barrier and consequently leaking into maternal circulation, depleting Hpx. We hypothesized thus that women with PE and SGA infant would have lower levels of Hpx compared to women with PE and non-SGA infant, because Hpx binds and detoxifies heme and HbF that is released from the feto-placental unit. Although the levels of plasma Hpx was lower in women with PE who gave birth to SGA infant compared to women with PE and non-SGA infant, the difference was not significant. Type II error is possible due to a small sample size. One woman in the group of women with PE who gave birth to SGA infant had exceptionally high plasma Hpx and A1M values and this increased variability substantially. This woman differed from the other in that group by having a medical history of chronic hypertension and thus superimposed PE and she was the only woman who received mini-ASA in the group of women with PE who gave birth to SGA infant.

Recent studies (Ferrazzi et al., 2018; Tay et al., 2018) suggest that women with PE and SGA or growth restricted fetus have a distinct cardiovascular phenotype characterized by lower cardiac output and higher peripheral vascular resistance. It is tempting to speculate that there is a connection between the impaired cardiovascular function and the decreased levels of heme scavenger Hpx. A possible explanation may be leakage of extracellular HbF of fetoplacental origin in maternal circulation. As a consequence, heme is released from metabolized extracellular HbF and depleting Hpx as recently shown (Anderson et al., 2018). Imbalance in the scavenging capacity causes vasoconstriction by reducing nitric oxide availability and impaired cardiac function. In fact, heme has been shown to induce contractile dysfunction in human cardiomyocytes *in vitro* (Alvarado et al., 2015). Furthermore, Hpx has been shown to have cardio-protective effect and it preserves systolic function by limiting heme-driven oxidative stress in mice (Ingoglia et al., 2017). Further studies, where cardiac function is evaluated simultaneously with the concentration of heme in maternal circulation, are needed to further clarify whether this suggested mechanism has clinical relevance.

In contrast to previous studies, we found increased plasma concentration of Hpx in PE women compared to low-risk controls. After excluding controls and PE women treated with mini-ASA, there was no statistically significant difference in the concentrations of Hpx between PE women and controls. In previous studies, where women treated with mini-ASA were not included, the concentration of Hpx was decreased, albeit marginally, in early pregnancy in women who later developed PE (Anderson et al., 2016). The concentration of Hpx in PE women has been shown to be even more decreased compared to controls just before delivery (Anderson et al., 2018). Thus, when focusing on women not treated with mini-ASA, our results suggest that Hpx depletion, due to heme-Hpx binding, is a continuous process, which starts slowly in early pregnancy and intensifies in third trimester when PE is manifested clinically. Further studies with a significantly larger patient group are needed if we try to detect the very marginal differences of the early stages of this process in order to clarify the dynamics of this process.

As regards to mini-ASA prophylaxis, 3 high-risk controls and 7 PE women who had participated in ASA trial were included in this study. Overall, these 10 women had significantly higher plasma Hpx concentration compared to controls and PE women who did not receive mini-ASA prophylaxis. Mini-ASA could possibly have an impact on Hpx concentration by improving placental perfusion. We have previously hypothesized that the up-regulation of HbF gene expression in the preeclamptic placenta may be induced by hypoxia caused by insufficient placental perfusion (Gram et al., 2015). Mini-ASA may improve the placental perfusion through inhibition of the potent vasoconstrictor Thromboxane A2 (TXA2) or by preventing placental thrombosis formation improving the blood flow in the PE placenta. Improved placental perfusion could then possibly prevent up-regulation of the HbF gene expression thereby reducing the leakage of HbF and heme into the maternal circulation. As previously shown, small amount of heme administered intravenously in rhesus monkeys increases the Hpx levels by increasing the rate of Hpx synthesis (Foidart et al., 1982). In contrast, large amount of heme has been shown to reduces Hpx levels by increased Hpx catabolism. Further studies are needed to understand the role of mini-ASA on Hpx dynamics.

## Conclusion

This study shows increased concentration of plasma A1M in high-risk normotensive pregnant women in late second trimester.

## Author Contributions

SH, BÅ, and HL conceived and designed the analysis. GK and KM performed the analysis and wrote the manuscript. KM, PV, KR, EH, EK, and HL collected the data and contributed the data.

## Conflict of Interest Statement

SH and BÅ holds patent related to diagnosis and treatment of preeclampsia and are co-founders of A1M Pharma and Preelumina Diagnostics (www.a1m.se). The pre-existing intellectual properties involve 4 patents owned by A1M Pharma;

1. HBF and A1M as early stage markers for preeclampsia-1550535
2. Medical use of A1M-2638915
3. Diagnosis and treatment of preeclampsia-201500335
4. Biomarkers for preeclampsia-PA 2015 70146

The remaining authors declare that the research was conducted in the absence of any commercial or financial relationships that could be construed as a potential conflict of interest.
