# Supplementary material for: Plasma Heme Scavengers Alpha-1-Microglobulin and Hemopexin as Biomarkers in High-Risk Pregnancies
Source: Front Physiol. 2019 Apr 4;10:300. doi: 10.3389/fphys.2019.00300 (PMC6458234; doi:10.3389/fphys.2019.00300)
Supplement: Supplementary file 1 [file Table_1.docx]

| **Inclusion** |
| --- |
| Obesity (body mass index over 30 kg/m2) |
| Chronic hypertension (≥140/90 mmHg or medication for hypertension before 20 weeks of gestation) |
| Sjögren’s syndrome |
| A history of Gestational diabetes |
| A history of pre-eclampsia (blood pressure ≥140 mmHg systolic or ≥90 mmHg diastolic and proteinuria ≥0.3 g/day or dipstick equivalent in two consecutive measurements) |
| A history of small for gestational age (birthweight < 2SD) |
| A history of fetus mortus (foetal death after 22 weeks of gestation or >500 g weight in a previous pregnancy) |
| Systemic lupus erythematosus  Type I diabetes mellitus |
| **Exclusion** |
| Tobacco smoking (during this pregnancy) |
| Multiple pregnancy |
| A history of asthma |
| A history of peptic ulcer |
| Placental ablation |
| Inflammatory bowel diseases (Crohn’s disease, colitis ulcerosa) |
| Rheumatoid arthritis |
| Haemophilia or thrombophilia (previous venous or pulmonary thrombosis or coagulation abnormality) |

Supplementary Table 1

Inclusion and exclusion criteria of the risk group.
